# Vitamin D Supplementation and Genetic Polymorphisms Impact on Weight Loss Diet Outcomes in Caucasians: A Randomized Double-Blind Placebo-Controlled Clinical Study

**DOI:** 10.3389/fmed.2022.811326

**Published:** 2022-03-03

**Authors:** Konstantinos Xenos, Maria Papasavva, Athanasios Raptis, Martha-Spyridoula Katsarou, Nikolaos Drakoulis

**Affiliations:** ^1^Nutrigenetics Department, Athens Euroclinic Hospital, Athens, Greece; ^2^Research Group of Clinical Pharmacology and Pharmacogenomics, Faculty of Pharmacy, School of Health Sciences, National and Kapodistrian University of Athens, Athens, Greece; ^3^X4nutrition LP company, Athens, Greece

**Keywords:** vitamin D, weight loss diet, obesity, 25(OH)D, VDR, adrenergic receptors, single nucleotide polymorphisms

## Abstract

Vitamin D deficiency or insufficiency is common in obese people, with some studies suggesting that low vitamin D level might be an independent predictor of obesity. Thus, the purpose of the present randomized, double-blind, placebo-controlled study was to investigate the effect of oral spray vitamin D_3_ 3000 IU supplementation along with personalized weight-loss diet on obesity markers in overweight and obese Caucasians with vitamin d deficiency or insufficiency. The impact of vitamin D receptor (VDR) and adrenergic receptors (ADRs) genetic variants on vitamin D levels and weight loss diet outcomes was also investigated. After signing informed consent, a total of 125 eligible volunteers were randomly assigned into vitamin D (vitamin D_3_ 3000 IU/d oral spray supplementation, *n* = 76) or placebo (xylitol, water, mint, *n* = 49) group following a weight loss program (600 calories less than the total energy expenditure of each volunteer) for 3 months. Fat mass, BMI, REE and 25(OH)D serum level were monitored on baseline and each month. DNA samples were extracted from buccal swabs and genotyped for the rs2228570 (*VDR*), rs1544410 (*VDR*), rs731236 (*VDR*), rs1800544 (*ADRA2A*), rs1801252 (*ADRB1*), rs1042713 (*ADRB2*), and rs4994 (*ADRB3*) polymorphisms. Statistical analysis was performed using SPSS package (v.23). Between group comparisons revealed significant improvement in serum 25(OH)D level and greater reduction in weight, BMI and fat percentage in the vitamin D group compared to placebo group (*p* < 0.05). In the vitamin D group, carriers of the rs2228570 T allele tended to have greater vitamin D level improvement compared with the homozygous C allele (*p* = 0.067). Furthermore, heterozygous (CT) for the rs731236 tended to have lesser weight loss (*p* = 0.068) and for the rs1042713, a lower decline in fat percentage was observed for homozygous AA carriers compared to the heterozygous (*p* = 0.051). In the control group, differences in weight loss (*p* = 0.055) and BMI (*p* = 0.045) were observed between rs1544410 AA and GG homozygous. In conclusion, vitamin D oral spray supplementation seems to improve vitamin D status and decrease obesity markers during a weight-loss intervention in overweight/obese Caucasians with vitamin D deficiency or insufficiency. Also, the results of the present study indicate that *VDR* and *ADRs* genetic polymorphisms seem to influence vitamin D supplementation response and obesity markers.

## Introduction

Vitamin D is a fat-soluble vitamin synthesized from 7-dehydrocholesterol in the skin after exposure to sunlight or obtained from diet and dietary supplements. Vitamin D comes in two forms; vitamin D_2_ (ergocalciferol) and vitamin D_3_ (cholecalciferol) (1, 2). Two sequential hydroxylations convert vitamin D into its biologically active form; 25-hydroxylation in the liver, which produces 25-hydroxyvitamin D_3_ (25 (OH)D_3_, calcidiol), the major vitamin D_3_ circulating form in the body used as a valid vitamin D status biomarker, followed by the second 1α-hydroxylation in the kidney, which converts 25(OH)D_3_ to 1,25 dihydroxyvitamin D (1,25 (OH)_2_D_3_, calcitriol) (3–6). 1,25(OH)_2_D_3_ is the most active vitamin D metabolite and a steroid hormone with multiple skeletal and extraskeletal biological roles, mediated by the vitamin D receptor (VDR), that controls over several hundreds of genes (7, 8).

Worldwide data indicate that the prevalence of hypovitaminosis D is a serious global health problem in all ages, even in countries with sun exposure throughout the year (9). Level of serum 25(OH)D below 20 ng/ml (50 nmol/L) are defined as vitamin D deficiency, whereas 21–29 ng/ml (525–725 nmol/L)serum 25(OH)D level as vitamin D insufficiency (10). Hypovitaminosis D is common in obese individuals, while recommendations for obese individuals suggest higher doses of vitamin D (10). In addition, BMI and fat mass are factors inversely related with 25(OH)D level (11–15).

An indirect association between obesity and vitamin D deficiency is possible due to the less sunlight exposure lifestyle of obese individuals (16). Moreover, obesity-related hypovitaminosis D could be attributed to the decreased bioavailability of the fat soluble vitamin D in the circulation of obese individuals due to the greater storage of vitamin D in fat tissues (15, 17, 18). Contrariwise, studies suggest that vitamin D may regulate body composition (19, 20), while a recent meta-analysis indicates that vitamin D supplementation in overweight and obese individuals may serve as a possible therapeutic option for weight l oss interventions (21). Low 25(OH) D level results in an increased parathyroid hormone (PTH) concentration which subsequently stimulates calcium influx into adipocytes and thereby promotes adipogenesis. Another hypothesis that supports the involvement of vitamin D deficiency in the pathophysiology of obesity is that 1,25(OH)_2_D and VDR are implicated in adipocyte differentiation (19, 22). Nevertheless, causality direction and the underlying mechanism are still uncertain.

Obesity is a complex multifactorial disease affected by genetic, environmental, socioeconomic, and behavioral factor confluence, which raises remarkably the risk of debilitating morbidity and mortality. Overweight and obesity prevalence is alarmingly increasing, affecting over one-third of the world's population (23, 24). By the year 2030, if current trends continue, up to 57.8% of the world's adult population will be overweight and obese, as estimated by Kelly et al. (25). Since obesity and vitamin D deficiency and insufficiency are progressively widespread (26, 27), uncovering the casualty direction between them and identifying vitamin D supplementation treatment that has a beneficial impact on obesity and obesity-related disorders remains a crucial area of investigation. Therefore, the purpose of the present study was to investigate the effect of oral spray vitamin D_3_ 3000 IU/d supplementation along with personalized weight-loss diet on obesity markers in overweight and obese Caucasians with vitamin d deficiency or insufficiency. In addition, the impact of genetic polymorphisms in VDR and adrenergic receptors (ADRs) in vitamin D levels and weight loss diet outcomes was also investigated.

## Materials and Methods

### Study Design

The present double-blind placebo controlled parallel group designed clinical study was conducted in “Athens Euroclinic” Hospital, Athens, Greece between January and April 2017. The study was reviewed and approved by the Ethics Committee of “Athens Euroclinic” Hospital and all volunteers provided signed informed consent.

Participants were primarily recruited through advertisements in “Athens Euroclinic” Hospital. The target population for the project included overweight and obese (BMI > 25 kg/m^2^) Southeastern European Caucasians with vitamin D deficiency (serum 25(OH)D_3_ <20 ng/ml) or insufficiency (serum 25(OH)D_3_ = 20–30 ng/ml) (10), aged 18–59 years. Exclusion criteria were pregnancy and lactation; being a professional athlete; participation in weight loss diet intervention 3 months before the study; taking medications e.g., Hydroxychloroquine and Cholestyramine that could influence D absorption; diabetes mellitus and other pathologies besides obesity and having increased sun exposure lifestyle.

**Figure 1:**
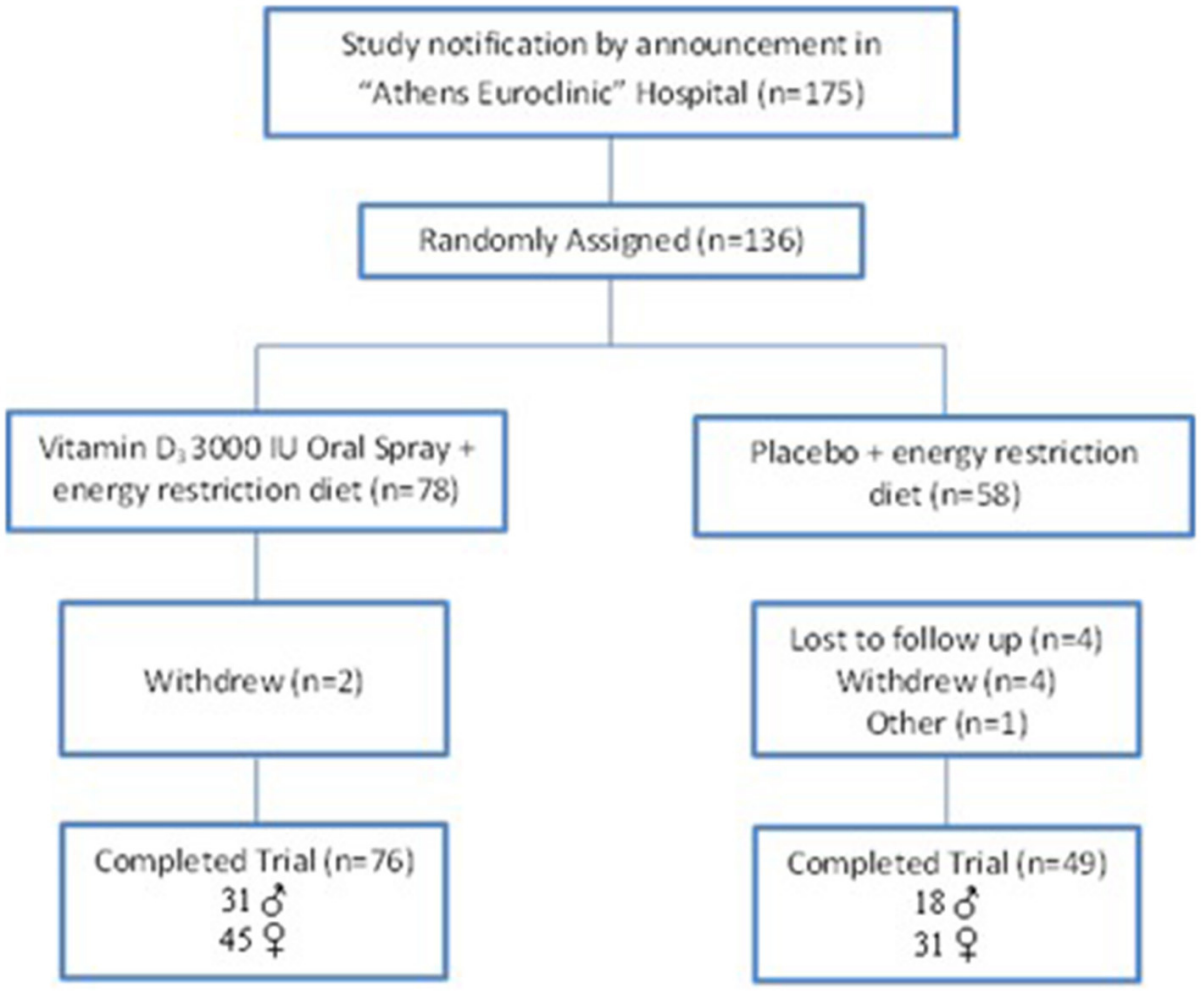
Flow diagram of the study.

Eligible volunteers were randomized into either weight loss program and vitamin D_3_ 3000 IU/d oral spray supplementation (Dlux 3000-Better You LTD) or weight loss program and placebo (oral spray containing xylitol, water, peppermint oil-Better You LTD) once daily, for 12 weeks. The goal of weight loss diet was a daily caloric restriction of 600 kcal less than the total energy expenditure of each volunteer, with reference points the Resting Energy Expenditure (REE) and the level of physical activity (PAL). Diet macronutrients were considered based on total caloric consumption as follows: 55 carbohydrates, 15 protein, and 30% fat. The Nutritionist Pro software (version 5.1.0, 2014, Axxya Systems, San Bruno, CA) enriched with recipes of the Greek traditional cuisine was used for weight loss diet design and analysis of dietary intake data and the energy and macronutrients' intakes calculation. Vitamin D intake from diet ranged from 170–250 IU/d. Participants were randomly assigned to each study group by the nutritionist, who was blind to the randomization status. Eligible volunteers had low level of physical activity (light walking). The design of the study is shown in Figure 1.

Subjects met the study dietitian at the initiation of the program, followed by monthly individual meetings for weight monitoring. Phone contact was performed on a weekly basis to assess the compliance with the study intervention, whereas compliance with the diet intervention was assessed with weekly food journals. Training of the subjects by an experienced dietitian using food model replicas was preceded. Each subject physical activity evaluation was carried out using smartphone's built-in applications provided by iOS and android operating systems. The primary outcomes of the study included changes in obesity markers (BMI, REE, %fat) and serum 25(OH)D level. Secondary outcome was the impact of *VDR* and *ADR* gene polymorphisms on obesity markers and serum 25(OH)D level changes.

### Measurements

Weight, height, REE, fat percentage, and serum 25(OH)D level measures were taken at baseline, whereas weight, REE, fat percentage and serum 25(OH)D level were measured after the intervention (12 weeks). Weight, REE and fat percentage were also calculated monthly. Weight and height were measured wearing only underwear, using the scale Tanita WB 110-A (Tanita, Tokyo, Japan) and Tanita HR200 Height Measuring Rod (Tanita, Tokyo, Japan), respectively. BMI was calculated as weight divided by the square of height (kg/m^2^). Individual total energy expenditure was estimated with indirect calorimetry method using Cosmed's FitMate (COSMED Srl Rome 3700041 Albano Laziale, Italia). Body composition was measured with Quadscan 4,000 device (Bodystat, Douglas, Isle of Man, UK).

Serum 25(OH)D levels were measured with LC-MS/MS (Liquid Chromatography with tandem mass spectrometry) using the Triple Quadrupole Mass Spectrometer (LC/MS/MS) from Agilent Technologies (Santa Clara, CA 95051, United States). LC-MS/MS vitamin D assays offer better accuracy at medical decision levels to correctly classify patients as vitamin-D deficient and sufficient.

Epithelial cells from the oral cavity of each participant were collected at baseline using sterile buccal swabs. DNA was extracted from the epithelial cells using commercial nucleic acid isolation kit (Tissue Nucleospin; Macherey-Nagel GmbH & Co., KG, Düren, Germany) and analyzed using the LightSnip kit (TIB MOLBIOL Germany) according to manufacturer's recommendations on the LightCycler 480 (LC480)-Instrument platform (Roche-Diagnostics, Mannheim, Germany). The following single nucleotide polymorphisms (SNPs) were analyzed: rs2228570, rs1544410, and rs731236 (Vitamin D Receptor -*VDR*), rs1800544 (Adrenergic Receptor Alpha 2A - *ADRA2A*), rs1801252 (Beta-1 Adrenergic Receptor - *ADRB1*), rs1042713 (Beta-2 Adrenergic Receptor - *ADRB2*), and rs4994 (Beta-3 Adrenergic Receptor - *ADRB3*).

### Statistical Analysis

Continuous variables were presented as mean ± standard deviation (SD) whereas categorical variables were presented as frequencies (*n*, %). Normal distribution of the data was tested by Kolmogorov-Smirnov test, thus parametric tests were performed. Post-intervention changes i.e., post-intervention minus baseline values, for each group were compared to examine the intervention responsiveness. Paired samples *t*-test was used for the assessment of intra-group mean values changes in continuous variables (serum 25(OH)D, weight, BMI, REE, fat percentage) before and after the intervention, while independent samples *t*-test was applied for between-groups differences. Pearson's chi-square (x^2^) test was performed for categorical variables (gender, distribution of genotypes). One-way ANOVA analysis was first applied to detect specific effects of the examined SNPs genotype status on serum 25(OH)D level, weight, BMI, REE, and fat percentage changes before and after the intervention. For the SNPs significantly associated (one-way ANOVA analysis *p* ≤ 0.05) with the examined variables, Bonferroni correction for multiple testing was then performed. The statistical power of the study was found to surpass 74.7% (β = 0.747). All statistical analyses were performed at a significance level of α = 0.05, using IBM (Athens, NTUA, Greece) SPSS Statistics Ver. 20.0 package.

## Results

In total, 136 overweight and obese Southeastern European Caucasians with vitamin D deficiency or insufficiency were initially recruited in the study. 125 participants completed the study, of whom 49 were men (39.2%) and 76 women (60.8%). The baseline characteristics of the study participants are given in Table 1. No differences in baseline characteristics between the groups were observed (Table 1). Within group analyses showed a statistically significant increase in serum 25(OH)D concentration (14.61 ± 4.86 vs. 34.09 ± 3.58, *p* < 0.001) in the vitamin D group. In addition, a statistically significant decrease in weight (103.02 ± 23.95 vs. 91.89 ± 22.19, *p* < 0.001), BMI (35.26 ± 7.32 vs. 31.44 ± 6.80, *p* < 0.001), fat percentage (37.54 ± 7.77 vs. 32.07 ± 7.61, *p* < 0.001), and REE (1604.76 ± 435.99 vs. 1486.03 ± 442.78, *p* < 0.001) was observed. Accordingly, in the placebo group, a much lower increase in serum 25(OH)D levels (14.16 ± 5.01 vs. 14.62 ± 4.78, *p* = 0.031) was observed, while a statistically significant decrease in weight (98.11 ± 21.94 vs. 88.50 ± 20.88, *p* < 0.001), BMI (34.00 ± 6.37 vs. 30.63 ± 5,99 vs. 32.07 ± 7.61, *p* < 0.001), fat percentage (38.04 ± 7.42 vs. 33.14 ± 6.79, *p* < 0.001), and REE (1536.59 ± 383.66 vs. 1416.69 ± 383.57, *p* < 0.001) was also observed. In between-group comparisons, statistically significant differences in serum 25(OH)D levels, weight, BMI, and fat percentage changes before and after the intervention were observed. Significant improvement in vitamin D status (0.46±1.45 vs. 19.49±4.66, *p* < 0.001) and reduction in weight (9.61 ± 2.91 vs. 11.13 ± 2.57, *p* = 0.003), BMI (3.36 ± 1.03 vs. 3.81 ± 0.85, *p* = 0.009), and fat percentage (4.90 ± 0.96 vs. 5.47 ± 1.03, *p* = 0.002) were observed in the vitamin D group compared to the placebo group (Table 2).

**Table 1:** Baseline characteristics of study participants.

**Variable**	**Placebo (*N* = 49)**	**Vitamin D (*N* = 76)**	***P***
Age (years)^*^	42.96 ± 11.07	40.14 ± 8.84	0.119^***^
Male, *n* (%)	18 (36.7)	31 (40.8)	0.650^**^
Female, *n* (%)	31 (63.3)	45 (59.2)	
Weight (kg)^*^	98.11 ± 21.94	103.02 ± 23.95	0.250^***^
Height (m)^*^	1.69 ± 0.08	1.70 ± 0.08	
BMI (kg/m^2^)^*^	34.00 ± 6.37	35.26 ± 7.33	0.327^***^
Body fat (%)^*^	38.04 ± 7.42	37.54 ± 7.77	0.721^***^
REE (kcal)^*^	1536.59 ± 383.66	1604.76 ± 435.99	0.373^***^
Serum 25(OH)D (ng/mL)^*^	14.16 ± 5.00	14.61 ± 4.86	0.624^***^

* *Values are mean ± SD*.

** *P-value for chi-square test*.

*** *P-value for Independent-Samples T-Test between means*.

**Table 2:** Serum 25(OH)D levels and obesity markers at baseline and after intervention.

**Variables**		**Placebo (*N* = 49)**	**Vitamin D (*N* = 76)**	P^*^
25(OH)D (ng/ml)	Baseline	14.16 ± 5.01	14.61 ± 4.86	0.624
	3 months	14.62 ± 4.78	34.09 ± 3.58	
	MD	0.46 ± 1.45	19.49 ± 4.66	**<0.001**
	% MD	5.23 ± 13.01	163 ± 106.57	**<0.001**
	P^**^	**0.031**	**<0.001**	
Weight (kg)	Baseline	98.11 ± 21.94	103.02 ± 23.95	0.250
	3 months	88.50 ± 20.88	91.89 ± 22.19	
	MD	9.61 ± 2.91	11.13 ± 2.57	**0.003**
	% MD^***^	−9.93 ± 2.63	−10.92 ± 1.97	**0.027**
	P^**^	**<0.001**	**<0.001**	
BMI (kg/m^2^)	Baseline	34.00 ± 6.37	35.26 ± 7.32	0.327
	3 months	30.63 ± 5.99	31.44 ± 6.80	
	MD	3.36 ± 1.03	3.81 ± 0.85	**0.009**
	% MD^***^	−9.93 ± 2.63	−10.92 ± 1.97	**0.027**
	P^**^	**<0.001**	**<0.001**	
REE (kcal)	Baseline	1536.59 ± 383.66	1604.76 ± 435.99	0.373
	3 months	1416.69 ± 38357	1486.03 ± 442.78	
	MD	119.90 ± 22.60	118.74 ± 21.32	0.772
	% MD^***^	−8.28 ± 2.63	−8.06 ± 2.95	0.674
	P^**^	**<0.001**	**<0.001**	
Fat Mass (%)	Baseline	38.04 ± 7.42	37.54 ± 7.77	0.721
	3 months	33.14 ± 6.79	32.07 ± 7.61	
	MD	4.90 ± 0.96	5.47 ± 1.03	**0.002**
	% MD^***^	−13.05 ± 2.21	−15.10 ± 3.71	**<0.001**
	P^**^	**<0.001**	**<0.001**	

*MD, Mean differences. Values are presented as mean ± SD*.

* *P-value for Independent sample t-test*.

** *P-value for Paired t-test*.

*** 100^*^(After-Baseline)/Baseline.

**Figure 2:**
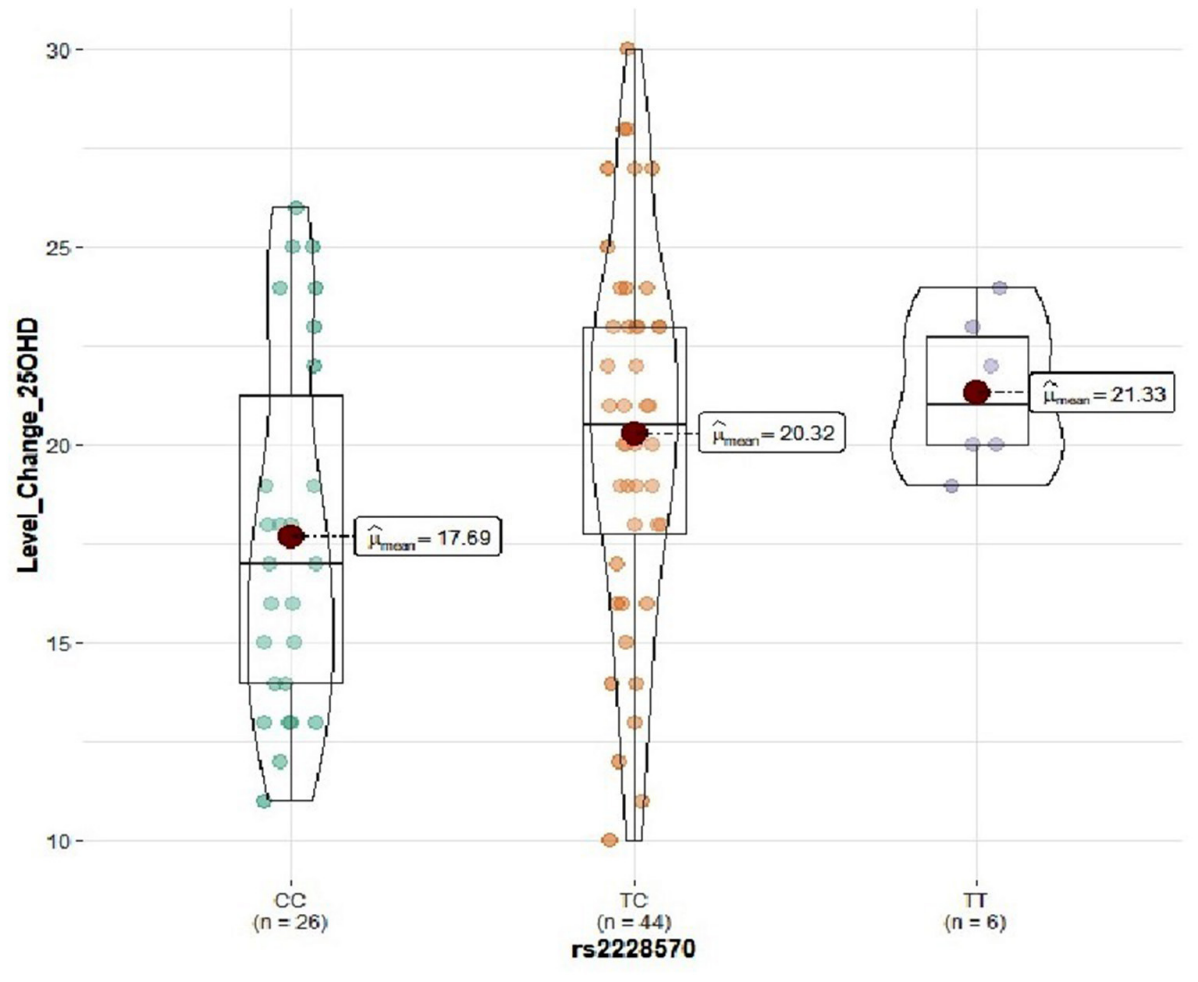
Violin plots displaying the mean 25(OH)D serum level change before and after the intervention in the vitamin D group according to rs2228570 genotype profile.

**Figure 3:**
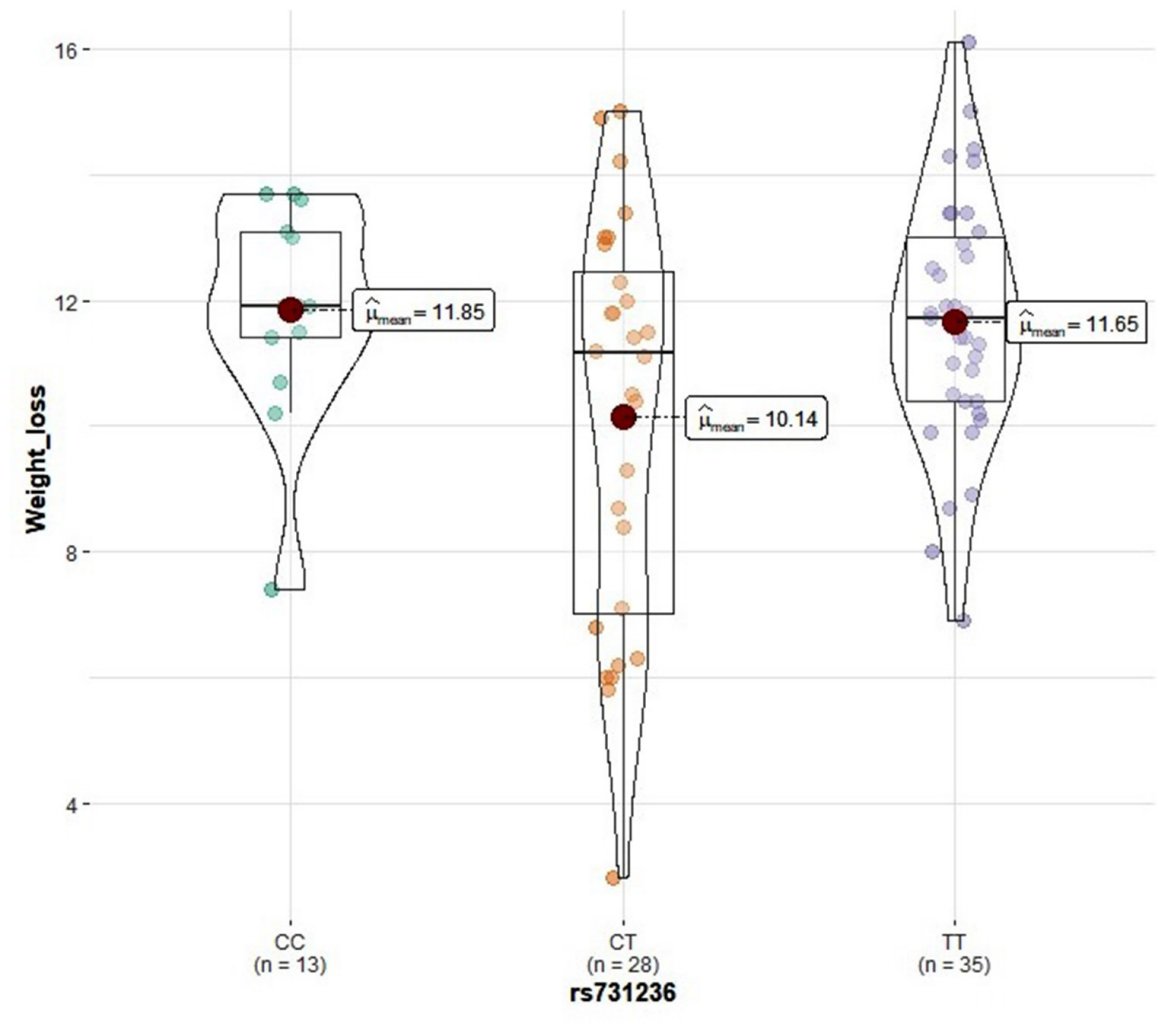
Violin plots displaying the mean values of weight loss in the vitamin D group according to rs731236 genotype profile.

**Figure 4:**
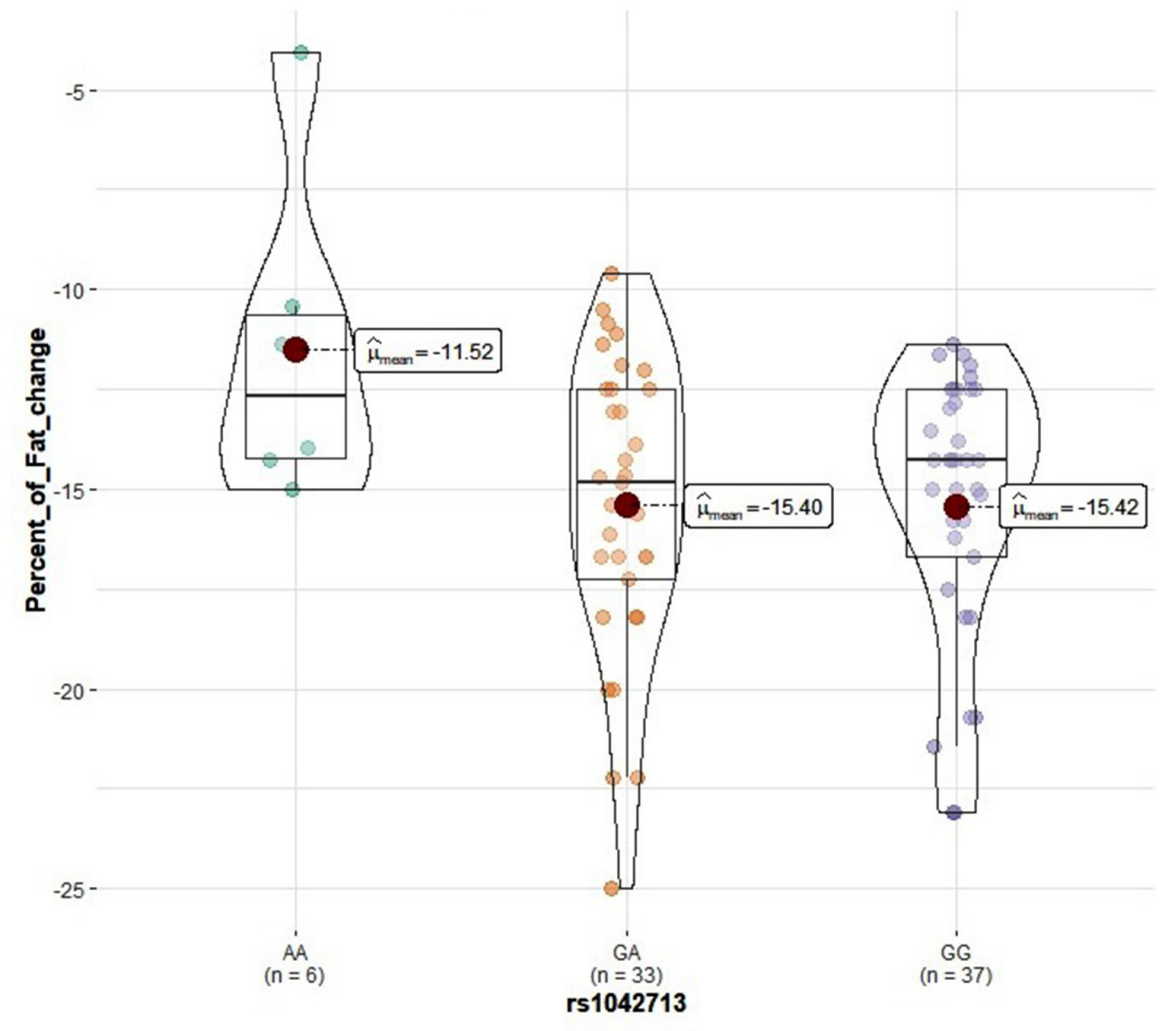
Violin plots displaying the mean values of fat percentage change before and after the intervention in the vitamin D group according to rs1042713 genotype profile.

**Figure 5:**
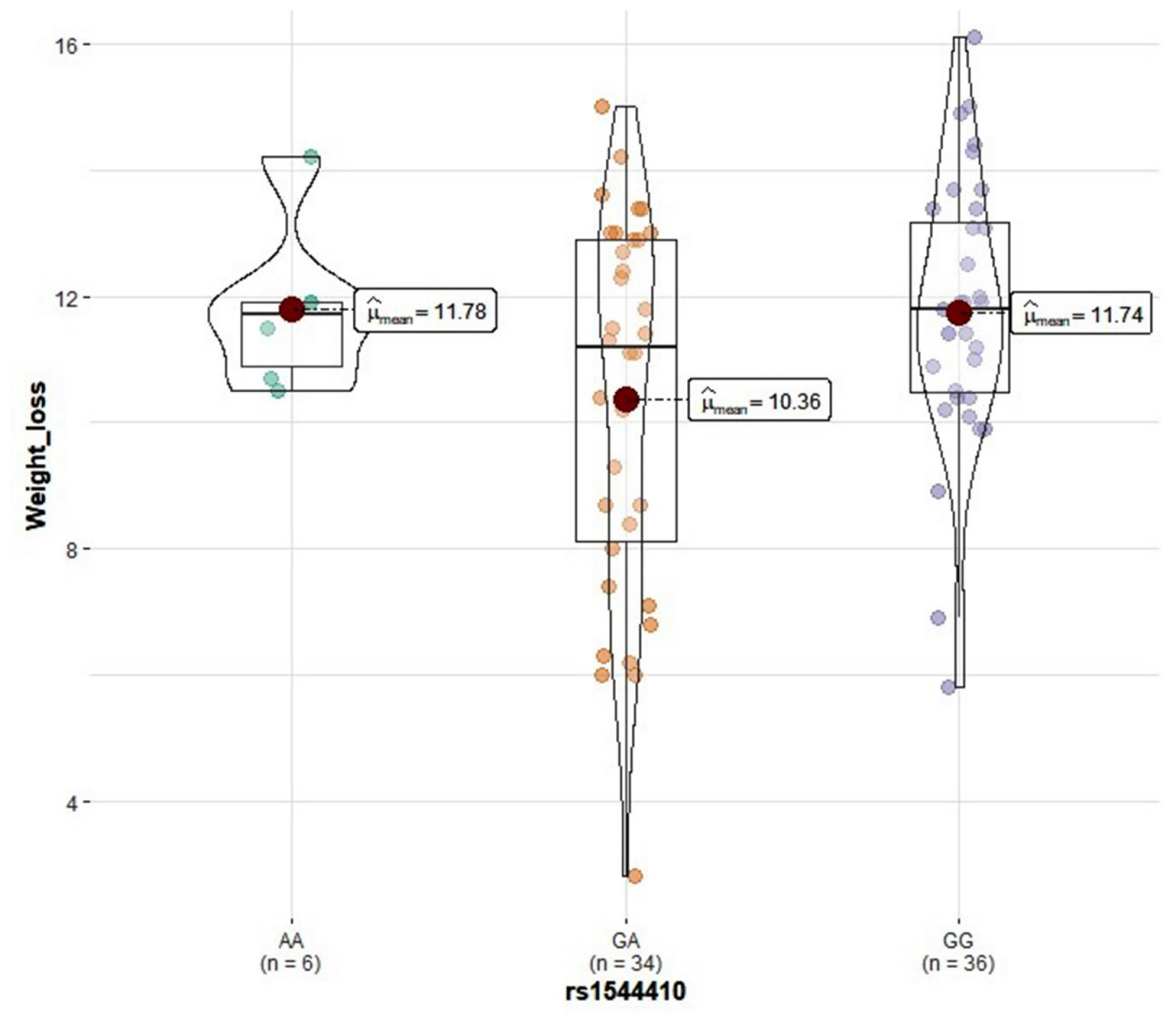
Violin plots displaying the mean values of weight loss in the control group according to rs1544410 genotype profile.

**Figure 6:**
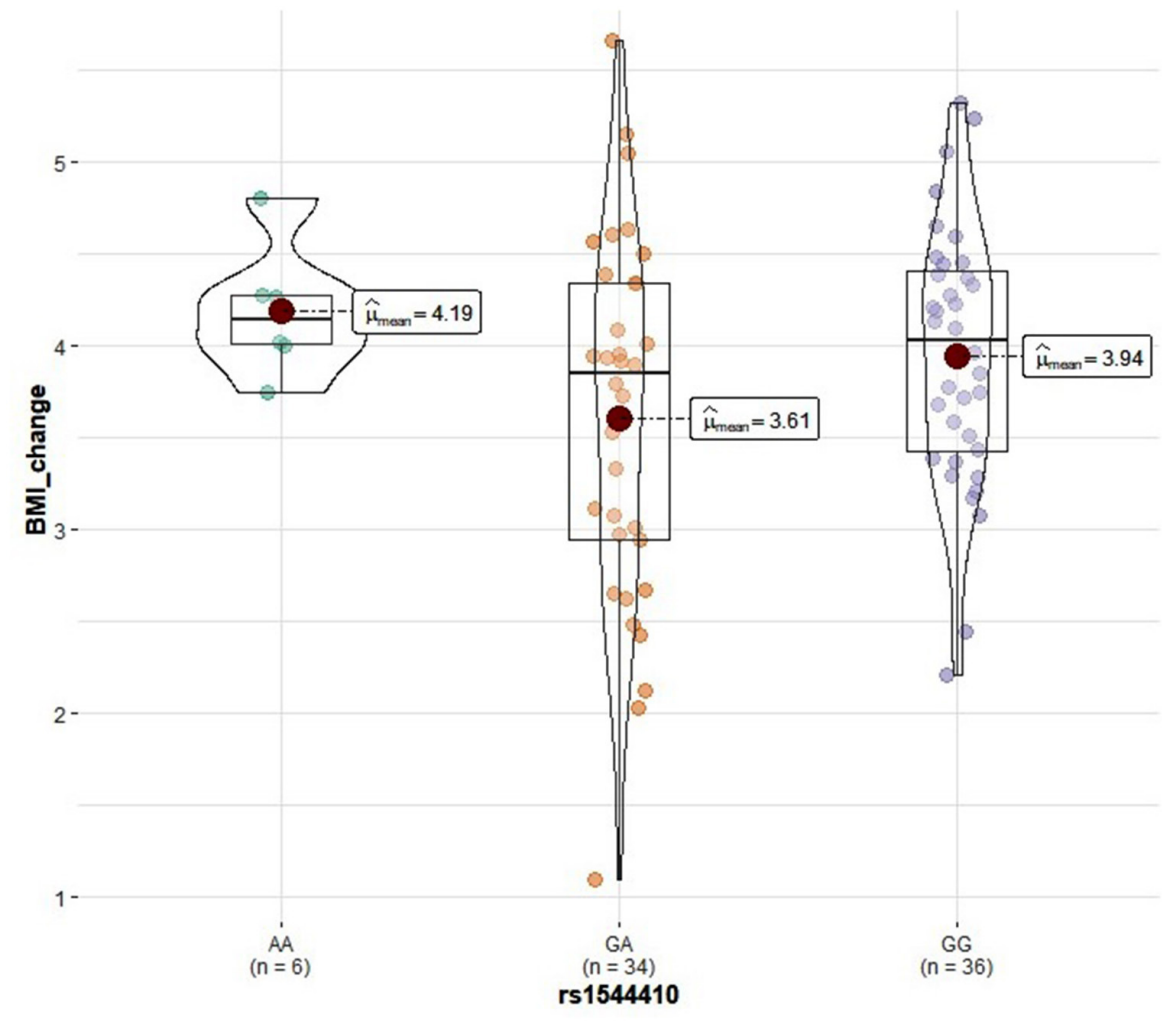
Violin plots displaying the mean values of BMI change before and after the intervention in the control group according to rs1544410 genotype profile.

Genotype distribution frequencies of rs2228570 (*VDR*), rs1544410 (*VDR*), rs731236 (*VDR*), rs1800544 (*ADRA2A*), rs1801252 (*ADRB1*), rs1042713 (*ADRB2*), and rs4994 (*ADRB3*) genetic polymorphisms in the study groups are shown in Table 3. No differences in genotype distribution frequencies were observed between the groups (Table 3). In the vitamin D group, carriers of the rs2228570 T allele tended to have greater vitamin D level improvement compared with the homozygous C allele (*p* = 0.067) (Figure 2). Furthermore, heterozygous (CT) for the rs731236 tended to have lesser weight loss (*p* = 0.068) (Figure 3) and for the rs1042713, a lower decline in fat percentage was observed for homozygous AA carriers compared with the heterozygous (*p* = 0.051) (Figure 4). In the control group, differences in weight loss (*p* = 0.055) (Figure 5) and BMI (*p* = 0.045) (Figure 6) were observed between rs1544410 AA and GG homozygous. In particular, homozygous for the G allele tended to exhibit better weight loss and statistically significant more reduction in BMI level compared with the homozygous for the rs1544410 A allele.

**Table 3:** Genotype distribution frequencies of *VDR, ADRA2A, ADRB1, ADRB2, ADRB3* gene polymorphisms in the study groups.

**Gene**	**SNP**	**Genotype**	**Placebo, *n* (%) *N* = 49**	**Vitamin D, *n* (%) *N* = 76**	P^*^
*VDR*	rs2228570	TT	1 (2.0)	6 (7.9)	0.293
		TC	27 (55.1)	44 (57.9)	
		CC	21 (42.9)	26 (34.2)	
	rs731236	TT	22 (44.9)	35 (46.1)	0.989
		CT	18 (36.7)	28 (36.8)	
		CC	9 (18.4)	13 (17.1)	
	rs1544410	AA	7 (14.3)	6 (7.9)	0.518
		GA	20 (40.8)	34 (44.7)	
		GG	22 (44.9)	36 (47.4)	
*ADRA2A*	rs1800544	GG	1 (2.0)	1 (1.3)	0.940
		CG	16 (32.7)	24 (31.6)	
		CC	32 (65.3)	51 (67.1)	
*ADRB1*	rs1801252	AA	43 (87.8)	69 (90.8)	0.447
		AG	5 (10.2)	7 (9.2)	
		GG	1 (2.0)	0 (0.0)	
*ADRB2*	rs1042713	AA	5 (10.2)	6 (7.9)	0.786
		GA	23(46.9)	33 (43.4)	
		GG	21 (42.9)	37 (48.7)	
*ADRB3*	rs4994	TT	43 (87.7)	66 (86.8)	0.881
		CT	6 (12.3)	10 (13.2)	

* *P-value for chi-square test*.

## Discussion

The results of the current double-blind placebo-controlled study in overweight and obese (BMI>25 kg/m^2^) Southeastern European Caucasians with vitamin D deficiency (serum 25(OH)D_3_ <20 ng/ml) or insufficiency (serum 25(OH)D_3_ = 20–30ng/ml), aged between 18 and 59 years, demonstrated that 12 weeks supplementation with vitamin D_3_ 3000 IU/d oral spray along with personalized calorie-restriction diet reduced significantly the mean of BMI and fat percentage while it significantly increased the level of serum 25(OH)D_3_compared with the control group. Nonetheless, no significant impact of the vitamin D oral spray supplementation was observed on REE.

According to the findings of this study, vitamin D_3_ 3000 IU/d supplementation of obese and overweight individuals with inadequate vitamin D status in combination with weight-loss program can potentially improve weight loss and reduce fat percentage. The results of the current study are in agreement with the study of Salehpour *et al*. which indicates that 12 weeks supplementation with vitamin D_3_ 1000 IU/d without a weight loss program in healthy overweight and obese women (*n* = 77) with mean serum 25(OH)D level 16.4 ± 12.56 ng/ml (41.8 ± 31.4 nmol/L), significantly decreased body fat mass (19). Similarly, Khosravi *et al*. found a significant reduction in weight, BMI and waist and hip circumference, while vitamin D level was improved in 50 overweight and obese women aged 20–40, in a 6-week 50000 IU/week vitamin D supplementation, following their usual diet (20). In addition, Lotfi-Dizaji *et al*. concluded that a 12 week 50,000 IU/week vitamin D supplementation along with calorie restriction diet in44 obese volunteers with vitamin D deficiency (25(OH)D <20ng/ml) diminished significantly weight and fat mass and improved serum 25(OH)D level (28).

Contrary to the previous findings, Sneve *et al*. reported that 20000 IU vitamin D supplementation once or twice a week for 12 months in 334 overweight and obese men and women without vitamin D deficiency, did not have any effect on weight changes, waist-to-hip ratio and body fat percentage (29). Zittermann *et al*., in a 12 months double-blind placebo controlled trialwith165 vitamin D deficient overweight or obese volunteers concluded that 3332 IU/d (83.3 μg) vitamin D_3_ supplementation in combination with a weight-reduction program did not adversely affect weight loss (30). In addition, Mason *et al*. in a 12-months randomized double-blind placebo-controlled trial found that 2000 IU/d vitamin D supplementation during a weight-loss intervention in overweight and obese postmenopausal women with vitamin D insufficiency had no effect on weight or fat loss (31). The main difference between these studies from those mentioned above is the longer period duration, which may affect the compliance of the volunteers. A meta-analysis of randomized controlled trials on the effect of vitamin D and calcium supplements on obesity concluded that taking vitamin D supplements had no effect on obesity markers (32).

According to a recent review article, obesity may represent an underlying confounding factor modifying the association between vitamin D deficiency and cardiovascular disease due to its increasing prevalence and strong correlation with cardiovascular disease and vitamin D status (33). Although the causal relation between vitamin D deficiency and obesity remains to be determined, the findings of the current study indicate that obesity may play a critical role in vitamin D deficiency. In particular, a statistically significant increase in vitamin D status was observed after the calorie-restriction diet intervention in the placebo group (14.16 ± 5.01 vs. 14.62 ± 4.78, *p* = 0.031). In agreement with this finding, Onal et al. found a negative relationship between >10% weight loss and vitamin D level, i.e. higher rise in vitamin D level as BMI decreased, although not statistically significant, due to the low number of samples (34). Also, the current findings indicate that an increase in serum 25(OH)D level from around 14 ng/mL to 34 ng/mL in combination with calorie-restriction program decreased weight, BMI, and fat mass, compared to the control group.

Both vitamin D status and obesity are under remarkable genetic influence, as reported by twin and family-based studies (35–37). Studies indicate that *VDR* gene polymorphisms are related to vitamin D levels, as *VDR* gene regulates vitamin D signaling pathways and vitamin D responsive genes, and may exert influence on adiposity and body composition (38–45). In the current study, the impact of three SNPs in *VDR* gene (rs2228570 (FokI), rs1544410 (BsmI), rs731236 (TaqI)) on the vitamin D status and obesity marker changes after the 3-month intervention was investigated. rs2228570 genotype status seems to influence vitamin D level in the vitamin D group, whereas rs1544410 and rs731236 genotype status was correlated with weight loss measures in control and vitamin D group, respectively. The effect of adrenergic receptor gene polymorphisms [rs1800544 (*ADRA2A*), rs1801252 (*ADRB1*), rs1042713 (*ADRB2*), rs4994 (*ADRB3*)] on obesity marker alterations after the intervention was also investigated, based on the major role of these receptor subtypes in the regulation of lipid mobilization and the correlation of these SNPs with obesity and metabolic syndromes (46–56). In the vitamin D group, rs1042713 genotype status seems to affect fat percentage changes, whereas no association was detected for *ADRA2A* rs1800544, *ADRB1* rs1801252, and *ADRB3* rs4994 polymorphisms.

*VDR* and *ADRs* genetic polymorphisms seems to influence weight-loss intervention outcomes. Consequently, identification of the most relevant genetic polymorphisms influencing weight-loss intervention outcomes could potentially improve dietary recommendations, advice and even drug therapy based on individual genetic susceptibility.

Strengths of the present study are the double-blind placebo-controlled design, the sufficient sample size, and the ethnic homogeneity of the study population. In addition, both genders were included in the study population, thus the results could be generalized in Southeastern European Caucasian population regardless gender and the occurrence of specific disease conditions. To our knowledge, the present study is the first to investigate the impact of genetic polymorphisms on obesity-related marker changes after weight loss diet and vitamin D supplementation.

The study has some limitation. Firstly, only one dose of vitamin D (3000 IU/d) supplementation was examined. Also, the effect of vitamin D supplementation on obesity markers was not examined independently, without a weight-loss diet intervention. The degree to which these findings can be generalized to non-Caucasian populations is not known. Finally, a possible limitation of the present study is not examining the potential role of additional biomarkers in vitamin D and obesity relationship.

In conclusion, 3-month vitamin D_3_ 3000 IU/d oral spray supplementation in overweight and obese Caucasian individuals with inadequate vitamin D status along with calorie-restricted diet program, improved 25(OH)D levels and contributed to a greater reduction in body weight, BMI, and fat percentage. Also, genetic polymorphisms seem to influence vitamin D supplementation response and obesity markers. Further, larger scale studies in Caucasian and non-Caucasian populations are required to validate the results of the present study.

## Data Availability Statement

The original contributions presented in the study are included in the article/supplementary files, further inquiries can be directed to the corresponding author/s.

## Ethics Statement

The studies involving human participants were reviewed and approved by the Ethics Committee of Athens Euroclinic Hospital. The patients/participants provided their written informed consent to participate in this study.

## Author Contributions

KX and ND: conceptualized and designed the study. KX: performed the study and reviewed and edited the manuscript. MP: drafted the manuscript. AR: performed the statistical analysis. M-SK: analyzed the samples. All authors read, reviewed, and approved the final version of the manuscript.

## Conflict of Interest

KX is the scientific director of the X4NUTRITION Company. The authors declare that this study received funding from X4NUTRITION & New Genomed LTD. The funders had the following involvement in the study: X4NUTRITION contributed by offering the active supplements (D3000 Better you) and the placebos. New Genomed LTD partially funded genotyping tests. The funders were not involved in the study design, collection, analysis, interpretation of data, the writing of this article or the decision to submit it for publication.

## Publisher's Note

All claims expressed in this article are solely those of the authors and do not necessarily represent those of their affiliated organizations, or those of the publisher, the editors and the reviewers. Any product that may be evaluated in this article, or claim that may be made by its manufacturer, is not guaranteed or endorsed by the publisher.

## Abbreviations

25(OH)D_3_: 25-hydroxyvitamin D_3_
1, 25(OH)_2_D_3_, 1: 25 dihydroxyvitamin D_3_
VDR: vitamin D receptor
ADRs: adrenergic receptors
PTH: parathyroid hormone
REE: Resting Energy Expenditure
SNPs: single nucleotide polymorphisms.
